# What Drives Saudi Gamers? A Study of Gender, Genre, and Geography

**DOI:** 10.3390/bs16020202

**Published:** 2026-01-30

**Authors:** Sultan A. Alharthi

**Affiliations:** Department of Software Engineering, College of Computer Science and Engineering, University of Jeddah, Jeddah 21959, Saudi Arabia; saalharthi8@uj.edu.sa

**Keywords:** video games, Saudi Arabia, gaming motivation, gender, genres, geography

## Abstract

The rapid expansion of gaming and esports in Saudi Arabia has prompted new interest in understanding how social and regional factors shape digital play. One trend is the variation in gaming preferences and motivations across gender and geographic regions. Previous research on gaming behavior has often centered on Western contexts and cross-national comparisons, overlooking how subcultural variation may shape players’ motivations and genre choices. Through a nationwide survey of gamers in Saudi Arabia, distributed via gaming community platforms, we examined how gender and region relate to gaming motivations, preferred genres, and intention to continue playing. Quantitative data were analyzed using inferential statistical methods, while qualitative responses were examined through thematic analysis to identify recurring patterns and contextual insights. Findings reveal that men are more likely to report performance and competition-driven motivations, while women show stronger preferences for escapism and casual gameplay. These results indicate that regional subcultures within Saudi Arabia shape gaming behavior, challenging generalized assumptions about player preferences. By demonstrating systematic regional differences in gaming motivations and preferences, this study extends games user research and informs the development of culturally responsive game design strategies.

## 1. Introduction

In recent years, digital gaming has become one of the most popular forms of entertainment across the globe. Countries across the Middle East and North Africa (MENA), including Saudi Arabia, are also experiencing a significant rise in gaming participation (Clément, 2019; Jarrah et al., 2023; NGES, 2022). Saudi Arabia now represents one of the largest gaming markets in the region, supported by widespread access to mobile technology, expanding internet infrastructure, and a highly engaged youth population (General Authority for Statistics, Saudi Arabia, 2023; Impact46, 2023).

In recent years, the Saudi government has introduced targeted national strategies to develop the country’s digital entertainment ecosystem, including substantial investments in game development, esports infrastructure, and international partnerships (NGES, 2022). These government-led initiatives have contributed to normalizing gaming as a mainstream activity, positioning it as both a creative industry and an economic opportunity (Impact46, 2023).

Gaming is deeply embedded in the everyday lives of Saudi youth, adults, and even older adults (Balahmar, 2024). According to recent data, over 90% of young Saudis report regular engagement with video games, making Saudi Arabia one of the most game-active populations in the region (Impact46, 2023). This trend is particularly significant given the country’s demographic profile: more than 70% of the Saudi population is under the age of 30 (General Authority for Statistics, Saudi Arabia, 2023).

Despite this high engagement, public perceptions of gaming in Saudi Arabia remain ambivalent. While gaming is increasingly recognized as a legitimate form of leisure and cultural participation, it still carries residual stigma, particularly when associated with excessive screen time, perceived unproductiveness, or men-dominated subcultures (Abolfotouh & Barnawi, 2024; Heining, 2020). As gaming becomes more integrated into daily life, it raises important questions about who plays games, what motivates them to play, and how those patterns differ across social and cultural contexts within a single country.

To understand how gaming behavior varies in Saudi Arabia, it is necessary to consider both individual and contextual factors. Gender is a particularly important variable in this setting, as cultural expectations and opportunities for men and women may shape their engagement with games in different ways (Lange et al., 2021; Lopez-Fernandez et al., 2019; Yee, 2016). At the same time, regional differences across Saudi Arabia may also influence the types of games people prefer and the reasons they play. While there is a growing body of research on gaming motivations and genre preferences, much of this work has focused on Western countries or cross-national comparisons (Lange et al., 2021; Ratan et al., 2021). Few studies have examined how these factors operate within Saudi Arabia, where cultural diversity exists across regions (AlKhamees, 2023; Balahmar, 2024). As such, little is known about how internal regional variation within Saudi Arabia affects gaming behavior.

This research addresses a clear gap in the literature by focusing on Saudi gamers and exploring how gender and subcultural variation influence gaming motivations and genre preferences. It moves beyond broad national comparisons to provide a more detailed understanding of how social and geographic factors shape digital play in a non-Western context. The findings of this study will contribute to a more complete understanding of the social and cultural dimensions of gaming and offer insights that may be useful for game designers and developers, game studios, policymakers, investors, and researchers interested in games, youth behavior, subculture studies, and media use in Saudi Arabia.

To guide this research, the study draws on motivational theory (Yee, 2016), which helps explain the psychological drivers behind player engagement, including competition, social connection, and creativity. It also incorporates perspectives from subcultural and regional analysis (Hofstede, 1998; Oyserman, 2017), emphasizing how local culture and geography shape gaming practices and preferences. Finally, the work references gendered construction theory (Cote, 2018) as a supplemental interpretive lens for understanding how gender norms may shape participation and identity in gaming spaces. This perspective supports, rather than directs, the empirical analysis of how gender and geography relate to gaming motivations in Saudi Arabia.

## 2. Literature Review

In recent decades, video gaming has emerged as a significant domain of entertainment, identity formation, and social interaction across the globe. As gaming has expanded demographically and culturally, researchers have sought to understand not just how much people play, but why they play and which types of games they gravitate toward (Yee, 2016). The psychological motivations behind gaming, such as competition, escapism, social interaction, and fantasy, have been extensively explored in international contexts (AlKhamees, 2023; Király et al., 2022; Lopez-Fernandez et al., 2019). These motivations often inform genre preferences, with players drawn to games that align with their psychological needs and lifestyle. For example, competitive players may favor action games, while casual gamers are more inclined toward adventure or role-playing genres (Greenberg et al., 2010b).

A key body of literature has advanced this field by examining how gender and cultural context shape gaming behaviors. Lopez-Fernandez et al. (2019) conducted an analysis of gaming profiles of women, highlighting how motivations and genre preferences are shaped by psychological and gender-related factors. Similarly, Ratan et al. (2021) compared gaming motives across Singapore, Germany, and the USA, revealing culturally specific genre and motivation patterns. These studies highlight that gaming is not merely a globalized activity but one deeply informed by local values, gender norms, and digital infrastructures. Despite the growth of this literature, players in the MENA region, particularly Saudi Arabia, remain significantly understudied. Recent data show that gaming in Saudi Arabia has shifted from a peripheral, often stigmatized hobby to a mainstream cultural practice, particularly among youth and young adults (AlKhamees, 2023; Almuhtadi & Alageel, 2025; Impact46, 2023; NGES, 2022). Gender and region continue to be strong structuring factors. Men gamers tend toward competitive and performance-based genres, women players are more often drawn to casual, narrative, or social play experiences (AlKhamees, 2023; Balahmar, 2024).

### 2.1. Characteristics of Gamers and Gaming

The term “*gamer*” has evolved significantly in recent decades, yet in many societies, including Saudi Arabia, the term continues to be shaped by enduring stereotypes, cultural norms, and generational assumptions. Before examining gaming behaviors and preferences across gender and region, it is important to first define what constitutes a “*gamer*” in both global and local contexts.

Traditionally, gamers have been imagined through a narrow lens: isolated, adolescent men, often portrayed as socially awkward, sedentary, and deeply immersed in solitary play on a console or PC (Williams et al., 2008). This image, while perhaps reflective of early gaming subcultures, has become increasingly outdated. Recent data show that video game players now span all age groups, income levels, ethnic backgrounds, and genders, with the global average age of a gamer estimated at around 33 years (Impact46, 2023; Newzoo, 2020).

One of the most persistent stereotypes is that gaming is inherently male. Although women now make up nearly half of gamers globally (Association, 2019), and Saudi Arabia has seen a marked increase in women’s participation in gaming (AlKhamees, 2023; Impact46, 2023). This perception has contributed to gendered divides in visibility and legitimacy: men are often publicly celebrated in esports and streaming platforms, while women may face skepticism, gatekeeping, or a lack of recognition within gaming communities. Importantly, these gendered dynamics do not imply uniform gaming practices across men and women, but rather shape the social contexts in which different forms of play are recognized and valued.

Another stereotype is that gaming is inherently isolating. However, much of contemporary gaming, particularly in Saudi Arabia, takes place in highly social contexts. Online distributed multiplayer games (Wuertz et al., 2018), streamed esports tournaments (Esports World Cup Foundation, 2024; Matsui et al., 2020), and co-located social gaming are common (Kappen et al., 2014). Far from being confined to solitary play in private spaces, Saudi gamers often use gaming as a medium for socialization, competition, and cultural expression (Arab News, 2018). This does not suggest that solitary or single-player gaming is inherently negative, but rather that social and solitary forms of play fulfill different motivational and experiential functions within gaming cultures. Moreover, the shift toward mobile gaming in Saudi Arabia has expanded what it means to be a gamer. No longer limited to expensive gaming consoles, mobile platforms have democratized access to digital play across income levels, ages, and gender boundaries. This broad accessibility challenges older stereotypes and invites a more nuanced understanding of Saudi gamers as a diverse, multifaceted community engaged in a wide spectrum of genres. Within this diversity, prior work suggests that women are often more likely to engage in socially oriented or communicative play contexts, while men are more visible in competitive or performance-driven games (Wohn et al., 2020).

Still, stigma persists, particularly around the idea that gaming is frivolous, addictive, or culturally inappropriate (Abolfotouh & Barnawi, 2024; Almuhtadi & Alageel, 2025; Heining, 2020). While such concerns are not unique to Saudi Arabia, they are shaped by specific societal expectations around productivity, gender roles, and media consumption. As Saudi Arabia positions itself as a future hub for esports and game development, the image of gamers is likely to continue shifting, from marginal figures to recognized participants in digital culture.

### 2.2. The Interplay of Gender, Genre, and Geography in Gaming Behavior

Understanding how gender influences gaming motivations and genre preferences has been a focal point of games user research, especially when contextualized within different cultural environments (Lange et al., 2021; Ratan et al., 2021; Wohn et al., 2020). Across this body of work, gender is generally treated not as a fixed biological determinant, but as a socially constructed category whose meanings and expectations vary across cultural contexts and gaming environments. Kahn et al. (2015) cross-cultural scale revealed that similar player types exist across regions, but their prevalence and dominant motivations differ depending on cultural variables such as individualism, gender norms, and gaming access. In a related investigation, Wohn et al. (2020) compared casual and competitive multiplayer games and found that gender not only predicted the intensity of certain motivations (e.g., achievement, social play) but also shaped the types of games participants were drawn to. Together, these studies assume that motivational dimensions are broadly shared, while their expression is shaped by social roles, norms, and access conditions rather than inherent preference alone.

Research comparing gamer identities across countries has echoed these trends. Ćwil and Howe (2020) explored differences between gamers in Poland and the United States, showing that genre preferences were strongly linked to both national identity and gender. U.S. players were more likely to express competitive motives, while Polish gamers showed stronger preferences for single-player and story-driven games, with these differences reflecting how shared motivational dimensions are mediated by national cultures and gender-role expectations rather than arising from culture-independent preferences. Similarly, Michelini et al. (2023) analyzed motivational profiles across seven countries and found that gender differences in motivations were stable across contexts, but their intensity varied by region.

Furthermore, Gackenbach et al. (2016) compared Chinese and Canadian gamers and highlighted that both gender and cultural identity affect genre preferences and gaming behavior, though not always in predictable ways. For instance, while Canadian female gamers preferred casual genres, Chinese female gamers leaned more toward strategy and RPGs, perhaps due to localized game offerings or differences in gender socialization. These findings highlight that genre preferences emerge not only from individual motivation but also from structural factors such as local market availability, platform access, and the cultural legitimacy of gaming practices within specific national contexts. This perspective is particularly relevant for interpreting the Saudi context, where genre popularity and play styles are shaped by a combination of motivational tendencies, cultural norms, and historically specific patterns of access and acceptance.

This complexity was reinforced by Lange et al. (2021), who challenged long-standing stereotypes about gender and genre, finding considerable overlap between male and female preferences once gaming frequency and access were accounted for. Meanwhile, Hilgard et al. (2013) proposed a psychometric approach to measuring motivation and preference, emphasizing that personality traits, gender identity, and gaming experience all shape how players engage with specific genres. Collectively, these studies assume that gaming motivation is multi-dimensional, culturally mediated, and socially situated, rather than biologically fixed or universally expressed.

Recent work on gamer profiles has highlighted how local culture, language, and infrastructural conditions influence gaming practices, access, and modes of participation, rather than fixed player traits (AlKhamees, 2023; Khoshsaligheh & Ameri, 2020). Furthermore, contemporary typology models provide further insight into how motivations and preferences shape gameplay (Vera Cruz et al., 2023). For example, the BrainHex model and the Hexad framework both help identify different gamer archetypes by capturing motivational and behavioral player patterns (Nacke et al., 2014; Tondello et al., 2016). Despite this wealth of research, a key limitation persists: Saudi Arabia remains absent from these frameworks. This omission is particularly striking given the country’s rich internal diversity. Administratively, Saudi Arabia is divided into 13 regions, Riyadh, Makkah, Madinah, Qassim, Eastern Province, Asir, Tabuk, Hail, Northern Borders, Jazan, Najran, Al-Baha, and Al-Jouf, each with its own distinct cultural, linguistic, and socio-economic characteristics (General Authority for Statistics, Saudi Arabia, 2023). These regional distinctions influence not only daily life and social norms but also leisure practices, including patterns of gaming motivation, genre preference, and social engagement, patterns that have been examined in other contexts through psychological and sociocultural lenses and that reshape how games function as social and cultural media. As such, understanding Saudi gamers requires moving beyond national averages to examine subculture dynamics. Yet most existing studies treat countries as culturally homogeneous units (AlKhamees, 2023; Balahmar, 2024), overlooking intra-national variation that may be just as consequential as cross-national differences. By investigating how regional location within Saudi Arabia interacts with gender to shape gaming behavior, this study addresses a gap in the literature and contributes to a more nuanced understanding of Saudi gamers.

### 2.3. Understanding the Profile and Motivations of Saudi Gamers

As the gaming industry rapidly expands in the MENA region, Saudi Arabia has emerged as a regional leader in entertainment, gaming, and esports. Despite this growth, empirical studies focusing on Saudi gamers have only recently begun to appear (Abolfotouh & Barnawi, 2024; Alhamoud et al., 2022; Al-Khamees et al., 2023; AlKhamees, 2023; Alkhashil, 2024; Almuhtadi & Alageel, 2025; Balahmar, 2024; Mrayati, 2024). These studies have tackled diverse topics ranging from gaming addiction and digital culture to gamer identity, mental health, and sociocultural policy. However, a comprehensive picture of how gender and regional context influence gaming motivations and genre preferences remains missing.

Balahmar (2024) highlights the desire of Saudi gamers to become “*famous gamers*”. This aspiration is closely tied to the visibility of gaming influencers and esports figures in Saudi popular culture, where social media platforms and streaming services offer new avenues for recognition. These results show that gaming is increasingly seen by youth not merely as a form of leisure, but as a legitimate pathway to social mobility and personal identity formation. AlKhamees (2023) shows that Saudi gamers identify with local themes and cultural archetypes, offering strong evidence of localized gaming preferences (Goaid Alotaibi & Tuhaitah, 2021). Furthermore, Almuhtadi and Alageel (2025) examined how Saudi gamers perceived the impact of gaming on their physical health, social lives, and personal development. Saudi gamers view gaming not only as an entertainment outlet but also as an activity that can pose risks to their well-being, particularly when it affects mental health or social balance.

Saudi gamers, like players elsewhere, actively participate in social digital spaces; however, in the Saudi context, gameplay often involves negotiating local cultural expectations in relation to global gaming identities (AlBathi, 2022). These practices, particularly the use of Arabic–English language-switching, highlight the social adaptability and bilingual fluency of Saudi gamers and reflect their integration into globally networked gaming cultures. Alamr (2019) and Al-Jifri (2017) both highlight how online social games enhance English language acquisition, with informal gaming environments offering immersive vocabulary exposure that complements formal classroom learning. Alqurashi and Williams (2017) shifted the focus to educators, exploring teachers’ own experiences with gaming. Their results indicate that many teachers not only understood gaming’s cultural power but also play games themselves. Our prior research shows that motivation and social influence play a key role in shaping engagement with digital platforms among older adults (Alharthi, 2025), a dynamic also relevant in gaming contexts. These findings suggest a broader adoption of digital platforms, including gaming, across different age and professional groups in Saudi Arabia.

However, social engagement does not come without risks. Recent research reveals a troubling prevalence of cyberbullying among Saudi gamers. AlJaffer et al. (2021) found that nearly one in five young gamers in Saudi Arabia had experienced online harassment, and that many of those affected exhibited symptoms of depression. Attiah et al. (2022) draw attention to the physical health risks associated with prolonged gaming, such as neck and back pain among Saudi gamers, adding another dimension to the holistic assessment of gaming’s benefits and drawbacks. Furthermore, Abolfotouh and Barnawi (2024) focused on the psychological aspects of gaming, particularly addiction. They measured the prevalence of gaming addiction among Saudi teens and found significant correlations between addictive behavior and factors such as time spent gaming and emotional dependence on gameplay.

On the other hand, Alhamoud et al. (2022) and Almutairi et al. (2023) document rising rates of Internet Gaming Disorder (IGD) (Ko, 2014), reinforcing the need to distinguish between healthy engagement and compulsive gaming, particularly as games grow more socially and emotionally immersive. Mrayati (2024) also explored gamers’ perceptions of monetization strategies in video games and found that the use of loot boxes was positively correlated with signs of gaming-related distress and lack of self-control. These findings suggest that while the Saudi gaming community is increasingly socially dynamic, it also grapples with digital safety challenges that demand closer attention from educators and policymakers (Al-Khamees et al., 2023; Almohsen et al., 2022).

Moreover, Baabdullah (2018) argues that enjoyment and social influence are key drivers of gameplay, providing evidence that gaming choices in Saudi Arabia are deeply social and shaped by local cultural dynamics. Building on this, Alkhashil (2024) explores how Saudi gamers engage with historical narratives in digital games, finding that players are not passive consumers but actively reflect on the portrayal of Arab and Islamic histories. His research highlights a growing demand for culturally sensitive game design that resonates with local values and heritage. Complementing these insights, Naji and Iwar (2013) demonstrate that digital games frequently inherit and reproduce global stereotypes, often reinforcing inaccurate representations and stereotypical narratives about Arabs and Muslims. These patterns of misrepresentation have significant cultural implications, especially in the absence of localized alternatives (Giuliana, 2023). In this context, Al-Batineh and Alawneh (2022) show that many Arabic-speaking gamers feel disconnected from games that rely on superficial or inaccurate translations, arguing that effective localization requires deeper cultural awareness, particularly around religion, gender norms, and regional identity markers.

Tabletop and card games (Rogerson et al., 2016) are also popular among Saudi gamers, reflecting both traditional and contemporary forms of social play. Games such as *Baloot*, *Jackaroo*, *Carrom*, *Dominoes*, and *Ludo* are commonly played in homes, cafés, and social gatherings, often serving as a means of bonding across generations and regions (Arab News, 2018; Gulf News, 2020; Hensley, 2014). Yet despite their widespread presence in everyday life, little research explores their social or cultural role. This lack of attention points to a broader gap in the literature around local, non-digital gaming practices in Saudi Arabia, particularly in how they relate to gender, tradition, and social life.

### 2.4. Frameworks in HCI and Games User Research

Theoretical frameworks in HCI explore how users interact with technologies, systems, and everyday things (Clemmensen et al., 2016; Drachen et al., 2018; Norman, 2013). In games user research, theory helps bridge empirical data and human experience, revealing not only what players do but why they do it. Frameworks such as Activity Theory (Alharthi et al., 2021; Clemmensen et al., 2016), Self-Determination Theory (Ryan et al., 2006), Flow Theory (Chen, 2007), and Uses and Gratifications Theory (Li et al., 2015) are frequently used to understand player behavior, motivation, and engagement.

For instance, Tondello et al. (2016) extends Self-Determination Theory to categorize player motivations into six user archetypes. This framework offers a structured lens for understanding how different player types engage with game systems and interactive experiences. Yee (2016) conceptualize gaming motivation through recurring psychological dimensions such as Action, Social, Mastery, Achievement, Immersion, and Creativity. These constructs are rooted in established psychological theories of competence, autonomy, and relatedness, and have been widely adopted in game user research to model player engagement.

On the other hand, cultural and geographic contexts strongly influence play practices and meanings. Canossa et al. (2024) demonstrate how regional patterns in player telemetry data reflect cultural clustering, while Wang and Mao (2013) show that online gaming behaviors mirror social and linguistic boundaries within China. Adapting this logic, gaming communities often reproduce and negotiate gender norms. Kaufman et al. (2019) illustrate how design interventions can mitigate gender stereotyping in games. Similarly, Greenberg et al. (2010a) examined how gender and age influence orientations toward video games, revealing distinct motivational and attitudinal patterns across demographic groups. Their findings highlight the importance of contextualizing player motivations within cultural and social structures, a focus this study extends by analyzing how gender and regional subcultures shape gaming behaviors within Saudi Arabia.

Overall, theoretical frameworks in HCI and game user research provide the interpretive grounding that connects player behavior with cultural, psychological, and social meaning. Such frameworks have long guided researchers’ conceptualization of engagement, motivation, and player experience. Building on this foundation, the current study employs motivational theory to capture player drives, subcultural and regional analysis to account for local and cultural variation, and gendered construction theory to help interpret how gender norms shape participation. Together, these perspectives situate the study within a rich theoretical history of HCI and games user research and provide a coherent foundation for analyzing how gender and geography interact to shape gaming motivations in Saudi Arabia.

**Summary.** While prior studies collectively demonstrate an evolving research landscape on gaming in Saudi Arabia, highlighting increasing engagement, cultural acceptance, and demographic shifts, none have comprehensively explored the intersection of gender, regional subcultures, gaming motivations, and genre preferences. This gap is particularly significant given the diversity of Saudi society and the rapid growth of its gaming industry. Understanding how these dimensions interact is essential for constructing accurate gamer profiles, informing culturally relevant game design, and guiding future research and government initiatives. Thus, this study directly addresses these gaps by investigating the following:

**1.**  **How gender and region affect gaming motivations in Saudi Arabia?**
**2.**  **How those motivations translate into genre preferences of Saudi gamers?**
**3.**  **How all of these factors relate to gaming practices in Saudi Arabia?**

By combining motivational theory (Foundry, 2015; Yee, 2016) with subcultural and regional analysis (Hofstede, 1998; Oyserman, 2017), and grounding the analysis in gendered construction theory (Cote, 2018), this study offers a unique within-country perspective to the broader field of games user research (Drachen et al., 2018) and cross-cultural studies.

## 3. Methods

### 3.1. Research Instruments

This study employed a quantitative survey-based methodology to investigate how gender and regional location within Saudi Arabia influence gamers’ motivations, genre preferences, and future gaming intentions. The research design was guided by Yee’s Gamer Motivation Profile Survey (Foundry, 2015; Yee, 2016), a validated framework that segments player motivations into six high-level factors: *Social, Action, Achievement, Mastery, Immersion*, and *Creativity*. These six core factors are derived from twelve mid-level constructs, which are in turn assessed through a set of 39 survey items (see Supplementary Materials). For the purposes of this study, the model was adapted to the Saudi context by translating all items into Arabic and adding a small number of open-ended questions to capture participants’ experiences in greater depth. The full instrument consisted of 39 Likert-scale questions grouped into 12 mid-level categories, such as Competition, Community, Fantasy, and Completion. Each pair of categories corresponds to one of the six higher-order motivational dimensions, enabling multidimensional analysis of player preferences. The study contributes novel empirical data to the broader cross-cultural literature on digital play by localizing a widely used global model (Foundry, 2015; Yee, 2016) to the Saudi Arabian context.

### 3.2. Participant Characteristics

To explore geographic variation within Saudi Arabia, the country’s 13 administrative provinces were grouped into five commonly referenced regional clusters: **Ḥijāzī** (Western), **Najdī** (Central), **Janūbī** (Southern), **Shamālī** (Northern), and **Sharqī** (Eastern). This regional classification reflects colloquial and cultural understandings of Saudi geography and enhances the interpretability and statistical robustness of regional comparisons (Royal Commission for Riyadh City, 2023).

A total of 397 Saudi nationals participated in the study, representing all five major cultural regions of Saudi Arabia (Table 1). The sample was predominantly female (62.2%), with male participants accounting for 37.8%. Most respondents were between 18 and 24 years old (42.8%), followed by those aged 35–44 (25.7%) and 25–34 (15.6%). In terms of education, roughly half of participants reported a bachelor’s degree as their highest completed level of education (50.1%), while approximately one-third reported high school as their highest completed level (33.3%). The participant pool reflected a balanced mix of gaming engagement levels, with casual gamers forming the largest group (53.4%), followed by hobbyists (25.7%) and serious gamers (20.9%). Nearly one in four participants reported playing six to seven days per week, indicating high engagement levels. Regionally, Western Saudi Arabia accounted for 35.0% of responses, followed by Central (22.4%), Eastern (18.1%), Southern (12.9%), and Northern (11.6%) regions, ensuring broad national representation across demographic and geographic lines.

### 3.3. Data Collection Procedures

Data were collected through an online survey distributed across multiple channels to ensure broad reach among Saudi gamers. The questionnaire was hosted on Google Forms and disseminated through gaming community platforms, university networks, and social media channels over a four-week period. Participation was voluntary, and respondents were informed of the study’s purpose and anonymity before proceeding. Only Saudi nationals were eligible to participate.

### 3.4. Reliability and Validity Measures

The survey instrument used in this study was based on the validated Gamer Motivation Profile developed by Yee (Foundry, 2015; Yee, 2016), which has been widely employed in games user research to measure six primary motivational dimensions. To ensure contextual and linguistic accuracy, the adapted version underwent a pilot test with a small group of participants to evaluate clarity and translation consistency. Feedback from the pilot phase informed minor revisions to wording and response formatting before full deployment. After all the data were collected, we screened them for completeness. Incomplete submissions and submissions from non-Saudi participants were excluded from analysis.

The study was conducted in accordance with the Declaration of Helsinki and approved by the Institutional Review Board of the University of Jeddah (UJ-REC-349) on 28 May 2025 Informed consent was obtained from all subjects involved in the study.

### 3.5. Data Analysis

Survey data were analyzed to examine how gender, region, and other demographic variables influence gaming motivations and genre preferences among Saudi gamers. The dataset included standardized measures of six motivational dimensions, Social, Action, Achievement, Mastery, Immersion, and Creativity (Foundry, 2015; Yee, 2016), alongside demographic and behavioral variables. After cleaning the data, descriptive statistics were computed to summarize demographic trends and motivational scores. One-way ANOVAs were used to test differences in motivational factors across gender, age, education, gamer type, and play frequency. Where significant effects emerged, Tukey post hoc tests were applied. To assess combined effects of gender and region, two-way ANOVAs were conducted. These analyses revealed main and interaction effects across several motivational dimensions. Significant findings were visualized through bar plots and interaction graphs, illustrating how Saudi gamers’ motivation varies across gender, genera, and geography. SPSS (version 31.0.0) was used for all qualitative analyses, following standard assumptions, testing, and reporting conventions.

In addition to quantitative analyses, open-ended responses were examined using reflexive thematic analysis (Braun et al., 2022). This process involved repeated reading, initial coding, and the development of themes that capture players’ narratives about identity, social dynamics, safety, and cultural expectations within gaming spaces. These qualitative insights contextualized and deepened the interpretation of the statistical patterns. Gender construction theory was referenced during analysis as a contextual lens to interpret how cultural norms may shape players’ gender-related responses.

## 4. Findings

To better understand what drives gaming preferences across Saudi Arabia, we examined six core motivational dimensions, Social, Action, Achievement, Mastery, Immersion, and Creativity, through a two-way ANOVA analysis, testing for main effects of Gender, Region, and their interaction. While most motivational traits were shared across groups, several revealed meaningful distinctions that speak to how identity and context shape the way Saudi players engage with games.

### 4.1. Demographics and Motivational Dimensions

The survey results indicate that the majority of participants (62.6%) reported playing video games more than one day per week. Only 37.4% of participants reported playing 0–1 day per week. This suggests that video gaming is a regular activity for most Saudi gamers, occurring multiple times throughout the week. The results also reveal that mobile gaming is the most popular platform among Saudi gamers, with 54.7% indicating they primarily play on smartphones or tablets. Console gaming follows closely, chosen by 48.3%, while PC gaming also maintains a strong presence at 43.8%. In contrast, handheld consoles like the Nintendo Switch are significantly less used, with only 9.8%, and virtual reality platforms represent a niche interest at 13.2%. These findings emphasize the dominance of mobile gaming in Saudi, while highlighting sustained engagement across console and PC gaming as well.

When asked about their preferred video game genres, participants most frequently selected Action (52.5%), Adventure (51.7%), and Multiplayer games (46.4%). Other popular genres included Puzzle (45.3%), Simulation (32.1%), and Open World games (31.7%). This distribution suggests that Saudi gamers tend to favor immersive and dynamic gameplay experiences that emphasize challenge, exploration, and social interaction. Also, a majority of participants (58.9%) indicated that they engage in board games or card games like *Baloot* and *Jackaroo*, while 41.1% stated they do not. This highlights the continued cultural relevance of traditional gaming forms within the broader landscape of gaming preferences.

We examined whether gamer type (Casual, Hobbyist, Serious) had a statistically significant effect on the six motivational dimensions. Each analysis compared mean scores across the three gamer categories for each motivational outcome. Across all six motivational dimensions, gamer type did not significantly predict motivational differences. This suggests that the labels “casual,” “hobbyist,” or “serious” do not meaningfully capture players’ deeper motivations for engaging with games, a finding that could challenge common assumptions in gaming discourse and design.

Education level also did not significantly influence participants’ gaming motivations. This suggests that the reasons why Saudis play games, whether for socializing, competition, immersion, or creativity, are not strongly associated with their formal educational background, reflecting gaming’s broad appeal as a leisure activity across diverse educational levels.

Furthermore, gaming frequency did not have a significant effect on motivation. These findings suggest that why people play games is not strongly related to how often they play, at least within the motivational categories studied. This reinforces the idea that casual and frequent gamers may be equally motivated by narrative, sociality, achievement, or creativity, challenging assumptions that more time spent implies deeper or different motivation.

Moreover, age did not have a significant effect on motivation. These findings suggest that the underlying reasons why individuals engage with games are remarkably consistent across age segments within the Saudi gamer population. Whether younger players in their late teens or older players in mid-adulthood, participants reported comparable levels of motivation across all dimensions. This pattern reinforces the idea that age is not a defining variable in shaping the psychological drivers of play, and that motivations such as immersion, sociality, achievement, and creativity are broadly shared across generational lines.

**Figure 1:**
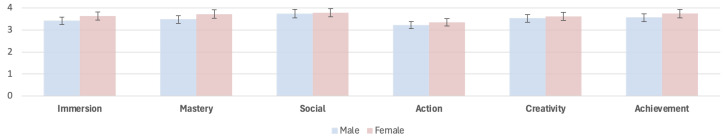
Mean scores for male and female participants across the six gaming motivation dimensions, with error bars representing 95% confidence intervals. Gender-based differences are most pronounced in Action, Social, Mastery, and Immersion motivations.

On the other hand, gender had a significant effect on two motivation dimensions: Mastery and Immersion (Figure 1). Specifically, a significant main effect of gender was found for Mastery motivation, F(1,395)=5.02, p=0.026, and for Immersion, F(1,395)=4.20, *p* = 0.041. These findings suggest that male and female participants differ in their tendencies to pursue skill development and narrative engagement, with male gamers likely exhibiting a stronger drive for mastery and female gamers showing higher immersion scores, aligning with gender patterns observed in prior gaming literature (Lopez-Fernandez et al., 2019). For the remaining motivation dimensions, Social (F(1,395)=0.15, p=0.700), Action (F(1,395)=1.09, p=0.296), Achievement (F(1,395)=2.47,p=0.117), and Creativity (F(1,395)=0.69,p=0.408), no significant differences were found. This suggests that motivations related to sociability, performance, and self-expression are largely shared between male and female gamers in the Saudi context. Overall, these findings point to gender-specific differences in how players relate to the depth and challenge of games, while reaffirming the universality of many core gaming motivations across genders.

### 4.2. The Interaction of Gender and Geography

A two-way ANOVA revealed a significant main effect of Gender on Social motivation, F(1,387)=6.89, p=0.009. No significant main effect of Region (p=0.625) or interaction between Gender and Region (p=0.363) was observed. This suggests that male and female gamers differ consistently in their social engagement with games, irrespective of geographic context. Female gamers are likely to report higher social motivation scores, aligning with previous findings that highlight their greater emphasis on connection and collaboration in gaming. The absence of regional effects supports the idea that social gaming operates as a cross-regional cultural practice in Saudi Arabia, unaffected by local variation.

A robust main effect of Gender was also found for Action motivation, F(1,387)=15.06, p<0.001. No significant effect of Region (p=0.850) or interaction (p=0.247) was detected. This confirms that gender is a major predictor of preference for fast-paced, dynamic gameplay, with male gamers generally showing stronger action-oriented inclinations. The consistency across regions indicates that gender-based patterns in gameplay style remain stable across diverse Saudi cultural landscapes, underscoring gender as a central factor in action-based gaming preferences.

The analysis revealed a significant main effect of Region, F(4,387)=3.11, p=0.015, as well as a significant Gender × Region interaction, F(4,387)=2.62, p=0.035. These results indicate that Achievement motivation is geographically patterned, but further, that this effect depends on gender. In certain regions, men may exhibit a higher drive for achievement through competition and goal completion, whereas in other regions, this difference may diminish or reverse. Such interaction effects underscore the importance of considering gendered cultural norms and opportunity structures across Saudi regions that may shape motivational profiles differently for men and women.

Mastery motivation also showed a significant effect of Region, F(4,387)=3.01, p=0.018, and a significant interaction with Gender, F(4,387)=2.61, p=0.035. The main effect of Gender was not significant (p=0.178). This suggests that regional variation plays a significant role in shaping players’ drive to master complex gameplay mechanics, but that the effect is not uniform across genders. In some regions, men may express stronger interest in skill development and strategic depth; in others, women may report comparable or even greater emphasis on mastery. These patterns reflect localized gaming subcultures that influence how skill-based engagement is gendered within region-specific gaming ecosystems.

The analysis revealed a significant main effect of Region, F(4,387)=3.81, p=0.005, and a newly significant Gender × Region interaction, F(4,387)=2.44, p=0.047. Gender alone did not show a significant effect (p=0.594). This updated result suggests that Immersion motivation, engagement with narrative, fantasy, and emotional depth, is shaped by both where a person lives and how their gender interacts with local gaming contexts. In some regions, immersion may resonate more deeply with female players; in others, male players may report higher narrative involvement. The significance of the interaction underscores the cultural embeddedness of immersive play, highlighting that motivations are not only personal, but also regionally and socially contextual.

Analyses revealed no significant effects for Creativity motivation, with all tests yielding *p*-values above (0.15). This reinforces the interpretation that the drive for creativity, customization, and expressive play is broadly shared across gender and regional lines in the Saudi gaming population.

### 4.3. Navigating Toxicity and Harassment

A recurring concern among participants centers on the emotional cost of online play, especially in mixed-gender or public multiplayer environments. While gaming is often positioned as a space for fun and escapism, these accounts complicate that narrative. One participant noted that *“gaming is not completely relaxing because there’s a lot of bullying and a slightly toxic community” [P3]*, reflecting the dual nature of the digital space, both recreational and hostile. The reluctance to play with strangers, as shared by another respondent, *“I don’t prefer playing with strangers” [P4]* highlight a broader sense of social vulnerability. Many participants described feeling unsafe or anxious, yet continued to play because of personal enjoyment: *“I don’t feel safe, but I play for fun.” [P2]*. This highlights a tension between personal agency and structural hostility.

Several participants recounted gendered harassment, including slurs tied to regional accents and stereotypical misogynistic phrases like *“go to the kitchen” [P3]*, especially when outperforming male players. These comments demonstrate not just isolated incidents but a patterned discourse that reinforces gender hierarchies, challenging the legitimacy and presence of women in competitive play. Also, insults based on local accents *“experience insults, slander, or bullying, for example, because of my accent” [P5]*, also illustrates how linguistic identity intersects with gender discrimination, revealing layers of marginalization. Collectively, these findings illustrate the precarious balance female gamers navigate: seeking enjoyment and community while having to constantly defend their right to participate and exist in these spaces. A recurring theme among Saudi female gamers is the preference for using text-only communication or remaining silent in multiplayer environments, a practice often rooted in social caution rather than technical limitation, *“I don’t like using a public microphone with people” [P1]*. The choice to conceal one’s face or real identity stems from concerns about online harassment and gender-based stereotyping. Voice becomes a minimal yet strategic tool, enough to participate, but not enough to expose. This pattern highlights the delicate balance female gamers maintain between engaging with online communities and protecting their social identities, reinforcing the idea that digital presence is often negotiated through layers of anonymity in gendered contexts.

For some participants, gaming functions as a form of escapism or emotional refuge, while for others it is a discreet form of self-expression. The home becomes both a sanctuary and a boundary, where women can explore expansive digital worlds but within strict social limits. This privacy, while protective, can also contribute to feelings of invisibility or marginalization within broader gaming and esports communities that thrive on visibility and public engagement. Despite growing participation, many Saudi female gamers still encounter various forms of gatekeeping that question their legitimacy in gaming spaces. These gatekeeping mechanisms range from subtle skepticism, such as being doubted as “real gamers”, to explicit exclusion from male-dominated online communities or tournaments. Cultural assumptions about gender and gaming amplify these barriers, reinforcing a hierarchical structure where male gamers are positioned as default authorities. This environment discourages open participation and reinforces the need for female-centered gaming spaces or platforms. The persistence of gatekeeping highlights the structural challenges women face in digital leisure, even as they develop skills and commitment equal to their male counterparts.

### 4.4. Influence of Culture and Local Context

Another key theme that emerged from the study revolves around family oversight and cultural contexts. Several participants highlighted the role of parents and their attitude toward gaming. They often have concerns about addiction and time management. One participant shared that *“they advise against excessive and addictive gaming,” [P5]* a sentiment that reflects common parental anxieties in Saudi households where screen time is often scrutinized. Similarly, another reported that *“they get annoyed by the number of hours I play,” [P1]* suggesting that gaming, while tolerated to an extent, may still be perceived as a wasteful activity when practiced excessively and remains stigmatized. In parallel, participants also expressed a desire for greater localization of global gaming content, particularly the inclusion of Arabic-language options. One noted, *“I wish there was an addition of Arabic to some of the most popular games,” [P3]* highlight the disconnect between the local player base and global game developers. This lack of linguistic and cultural representation can create a subtle sense of exclusion, reinforcing the idea that these games are not fully “for us”. The call for Arabic localization is not merely about convenience; it signals a demand for recognition, inclusion, and cultural legitimacy within global gaming ecosystems. Together, these findings point to the importance of both domestic cultural expectations and broader market structures in shaping the gaming experiences of Saudi female gamers.

### 4.5. Regional Patterns in Gaming Preferences Among Saudi Gamers

**Figure 2:**
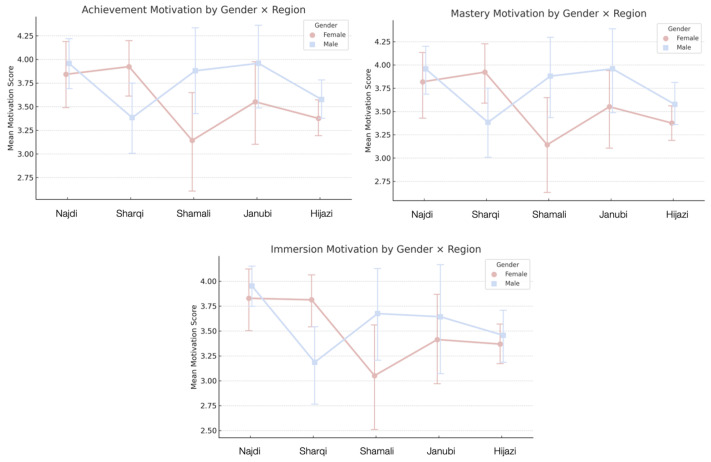
Interaction plots illustrating mean scores for male and female participants across Saudi regions for three motivational dimensions: Achievement, Mastery, and Immersion. The plots reveal regionally variable gender differences.

**Figure 3:**
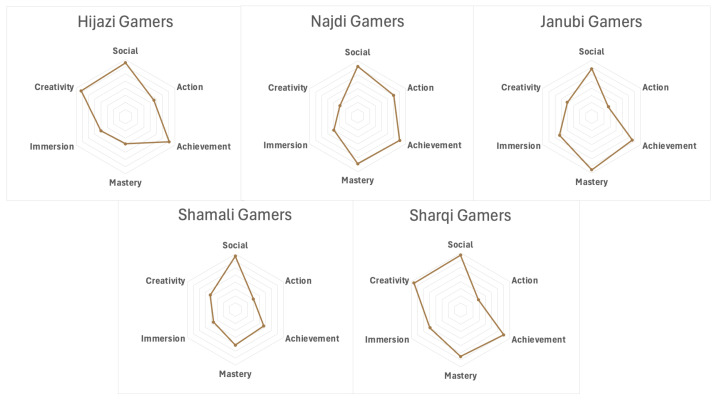
Radar charts illustrate the composite motivational orientations of gamers from the five Saudi regions across the six core dimensions of the Gamer Motivation Framework.

In mapping the landscape of gaming in Saudi Arabia, the data suggest that players from different regions vary in the kinds of games and gameplay elements they tend to gravitate toward. While these patterns should not be interpreted as fixed categories, they highlight distinctive constellations of preferences, aesthetic tendencies, and modes of engagement across regions (Figure 2 and Figure 3). It is important to note that these regional differences are modest in magnitude. Across all regions and genders, mean scores for motivational dimensions generally fall between 3 and 4 on the scale, indicating broadly shared levels of engagement rather than sharply divergent profiles. The patterns discussed here, therefore, reflect relative emphases and tendencies within a largely common gaming culture, rather than discrete or mutually exclusive regional styles of play.

Hijazi participants often gravitated toward genres that emphasize story, character development, and world-building, indicating a strong appreciation for narrative complexity and emotional engagement. Players from this region frequently described games as literary or cinematic experiences that offer cultural resonance and psychological immersion. Najdī participants tended to focus on structured challenge and strategic depth. Games involving tactics, competition, and performance-based objectives, such as real-time strategy, shooters, or puzzle-based games, were commonly favored. These players emphasized mastery, mental agility, and goal-oriented play, often highlighting efficiency, leadership, and progression as core aspects of their gaming experience.

Janūbī participants were often drawn to social and community-based play, favoring multiplayer games, cooperative storylines, and participatory game cultures. Many emphasized connection, playfulness, and shared experience. Aesthetic and emotional appeal were central, aligning with games that enable identity expression, customization, and light-hearted engagement. Shamālī participants showed interest in exploratory and reflective modes of gaming. Slower-paced, survival-themed, or resource-focused genres were frequently mentioned. These players often preferred open-world or sandbox formats that reward persistence and gradual progression, valuing observational playstyles marked by adaptability, endurance, and environmental awareness.

Sharqī participants frequently demonstrated interest in high-intensity, fast-paced games. Competitive titles, racing games, esports-oriented formats, and action platformers were commonly cited. Participants from this region described enjoyment of rapid decision-making, high-stakes play, and mechanics that rely on reflexes and precision.

Across all regions, these patterns are neither mutually exclusive nor strictly confined to geography. Instead, they represent evolving tendencies shaped by cultural heritage, digital media exposure, personal preference, and regional dynamics. Recognizing this diversity provides a clearer foundation for examining how gender, genre, and geography intersect and for situating Saudi gaming practices within a broader sociocultural context that reflects variation rather than homogeneity.

**Summary.** These findings provide a layered view of how gender and region influence gaming motivations in Saudi Arabia. While Social and Action motivations differed consistently by gender, with females favoring social play and males preferring competitive action, Achievement, Mastery, and Immersion were shaped more by regional context and gender–region interactions. These patterns suggest that motivational preferences are not merely the result of individual tastes but may reflect broader socio-cultural expectations and access to different forms of gaming content and communities. The stability of these gender differences across regions further implies that certain motivational dispositions may be culturally reinforced and embedded within prevailing norms around play and digital engagement. This highlights that gaming preferences are not only personal but also socially and culturally embedded. Creativity showed no significant differences across gender or region, pointing to a widely shared appreciation for expressive and imaginative play. This shared motivation may reflect a broader cultural resonance with open-ended gameplay and user-driven creativity across Saudi gamer communities. Overall, the results move beyond a monolithic view of the Saudi gamer and advocate for a multidimensional framework, one that accounts for identity, locality, and cultural nuance. These insights hold important implications for culturally responsive game development, targeted community engagement, and future research into regional subcultures of play.

## 5. Discussion

This study set out to explore how gender and regional identity shape gaming motivations within the Saudi context, using a localized application of Yee’s Gamer Motivation Model. By analyzing motivational dimensions across diverse sociodemographic groups and cultural regions, the research provides new insights into the complex interplay between individual identity and sociocultural environments in shaping digital play. In this section, we interpret the key findings through the lens of existing literature on gaming motivations, gender, and cultural variation. We consider how the results affirm or challenge global trends, and where they reveal context-specific dynamics unique to Saudi Arabia.

### 5.1. Saudi Gamers Are Social Beings

Saudi gamers are not isolated hobbyists but socially motivated digital participants, using gaming not just for entertainment, but as a meaningful vehicle for connection, collaboration, and cultural expression, aligning with research that highlights the inherently social nature of Saudi gamers (AlKhamees, 2023). Within the broader game studies literature, this social orientation corresponds to the Social dimension of Yee’s Gamer Motivation Model, which frames player engagement as driven by interaction, cooperation, competition, and relationship-building in digital play environments.

The data strongly suggest that Saudi gamers are fundamentally social in their gaming preferences. A substantial majority of participants (79%) expressed moderate to high enjoyment of social gatherings within games, with nearly 40% giving it the highest possible rating. This indicates that online multiplayer experiences, cooperative gameplay, and virtual communities form a central pillar of gaming culture in Saudi Arabia and how such players use games as social spaces across distributed games (Wuertz et al., 2018). Importantly, this pattern mirrors findings from international studies using Yee’s framework, suggesting that core social gaming motivations observed globally are also present in the Saudi context rather than being culturally idiosyncratic.

This finding aligns with the growing popularity of socially immersive titles in Saudi Arabia (e.g., Call of Duty, Fortnite, PUBG, Among Us), which offer spaces for both play and social interaction. Moreover, the emphasis on social features may be partly attributed to cultural values of community and shared experience, as well as the role of games as a safe medium for interpersonal engagement across gender and geography, a pattern also observed in studies documenting how Saudi gamers navigate family norms and social expectations around digital leisure (Almuhtadi & Alageel, 2025). Here, the Saudi context does not replace existing motivational theory, but rather shapes how socially driven play is enacted, highlighting the role of culture in mediating otherwise widely observed gaming motivations.

This pattern highlights a key driver of gaming behavior in Saudi Arabia: games are not merely personal pastimes but shared cultural experiences. The popularity of multiplayer and cooperative genres such as first-person shooters (FPS), sports games, and battle royales is consistent with this trend. These genres inherently encourage team-based strategy, voice communication, and competitive camaraderie, mechanics that appeal to a socially motivated audience. From a theoretical standpoint, this genre alignment illustrates how motivational structures translate into observable play practices, strengthening the link between abstract motivation models and empirical genre preferences.

The trend also reflects broader shifts in how younger Saudis, especially those aged 18–34, who make up the majority of this dataset, build and maintain relationships. With increasing access to global gaming platforms and communities such as Discord and Twitch, many Saudi gamers are not only engaging locally but also participating in international gaming cultures. This aligns with existing literature on gaming as a medium of transcultural socialization (Gackenbach et al., 2016) and supports findings that Saudi gamers frequently operate at the intersection of local identity and global digital networks (AlBathi, 2022). Consequently, the findings suggest that Saudi gamers exemplify a localized expression of shared global gaming motivations, contributing to ongoing debates in game studies about universality, cultural specificity, and the cross-cultural applicability of motivational models.

### 5.2. The Surprising Gaming Profile of Saudi Women

One of the most compelling outcomes of this study is the emergence of a robust and multifaceted gaming profile of Saudi women, challenging long-standing assumptions about gender and gaming in the MENA region, and aligning with earlier critiques of stereotypical “male gamer” narratives (Williams et al., 2008). Far from being peripheral participants, Saudi women are not only present in the gaming space, but they are also reshaping it. Their presence is quantitatively significant and qualitatively complex, revealing patterns of play that are socially collaborative, emotionally immersive, and expressive in character, echoing global findings that women often prioritize relational and immersive dimensions of play (Williams et al., 2008; Wohn et al., 2020).

Historically dominated by men, the region’s gaming culture is now becoming increasingly gender-diverse, a reflection of broader social transformations occurring in Saudi Arabia, including expanded digital freedoms, the rise of online communities, and evolving norms around women’s participation in public and virtual life, trends also documented in emerging studies of Saudi gaming cultures (AlKhamees, 2023; Balahmar, 2024). Women participants were most likely to identify as casual gamers (53%), compared to only 22% of men. However, this self-description does not imply disinterest or passivity. In fact, many women in the casual category reported regular engagement (over 2–3 days per week), with a wide variety of genres and motivations. This self-labeling may be culturally coded, aligning with modesty norms or resisting the perceived competitiveness associated with “serious” gaming identities, which is consistent with prior research showing that women often downplay gaming intensity due to social gatekeeping (Lopez-Fernandez et al., 2019; Wohn et al., 2020).

The prevalence of casual self-identification can thus be seen as a form of strategic gender performance, not a reflection of lesser interest. It also reflects cultural gatekeeping in gaming spaces, where women may adopt more socially acceptable gaming identities while still engaging deeply with the medium, paralleling findings on gendered identity management in online games (Ratan et al., 2021). The high frequency of multiplayer games and open-world games highlights the importance of collaborative play and narrative freedom. These genres offer space for social interaction, exploration, and character personalization, key features valued by Saudi women and consistent with findings from prior studies that show women are especially motivated by customization and socially rich game experiences (Van Reijmersdal et al., 2013).

Contrastingly, genres associated with rules-based mastery (e.g., simulation, strategy) were more common among men. This aligns with gender schema theory, which posits that societal cues shape the development of gendered preferences and competencies, a pattern also observed in cross-cultural analyses of genre preferences (Lange et al., 2021). Saudi women are interested in game genres that allow creativity, social interaction, and experimentation with identity, rather than just dominance.

Saudi women prioritize psychological and emotional engagement, often through immersive narratives and character or world design. Customization in particular can be read through the lens of digital selfhood, where avatars and game environments become tools of autonomous identity expression, especially vital in contexts where physical self-expression may be culturally restricted, an observation consistent with studies on expressive play and identity construction (Lopez-Fernandez et al., 2019). Interestingly, the relatively high challenge scores also defy the stereotype that women are less competitive. Instead, what emerges is a balanced profile: women are invested in both challenge and storytelling, but they often refuse the narrow logic of domination that defines “hardcore” gamer status in traditional discourse, echoing evidence that genre stereotypes fail to capture actual motivational diversity (Lange et al., 2021). The enjoyment of social gathering in games was rated highly by over 70% of women participants, affirming that gaming is a social bridge, not an isolated escape. In the Saudi context, where public gender mixing is culturally regulated, digital play offers a parallel world for social experimentation and community building.

Moreover, women frequently described gaming as a form of cultural negotiation, navigating between global digital norms and local values. This reflects the theory of cultural hybridity (Bhabha, 2015), where individuals form identities at the crossroads of tradition and modernity. For many Saudi women, gaming is not just a pastime but a cultural act, one that blends resistance, expression, and belonging. These findings call for a redefinition of what it means to be a gamer in the Gulf. Saudi women are neither rare exceptions nor passive consumers. They are active agents who engage with games on their own terms, embracing challenge, collaboration, narrative, and aesthetics in ways that defy monolithic gender assumptions. By occupying this space, Saudi women are not only redefining regional gaming cultures but also reshaping global gaming narratives, demanding recognition, representation, and equity within virtual worlds, an argument consistent with broader calls for more inclusive and culturally grounded game design (Al-Batineh & Alawneh, 2022). The data reveals a surprising gaming profile of Saudi women: socially driven, emotionally immersed, creatively expressive, and strategically positioned within a complex cultural terrain. They are not outliers; they are frontliners in the transformation of gaming culture in Saudi Arabia.

### 5.3. Gaming as Regional Identity

The findings of this study highlight how regional identity in Saudi Arabia plays a meaningful role in shaping the ways individuals engage with digital games, aligning with prior work showing that Saudi gaming cultures are shaped by local subcultural norms (AlKhamees, 2023; Balahmar, 2024). While gender remains a well-documented influence in gaming research (Lange et al., 2021; Ratan et al., 2021), this work reveals that geographic and cultural locality also inform motivational patterns and gameplay preferences, echoing cross-cultural findings that player motivations vary across sociocultural environments (Canossa et al., 2024; Wang & Mao, 2013). Regional context should therefore not be understood as a mere backdrop, but as an active site of cultural production that shapes how games are interpreted, enjoyed, and embedded within daily life, which is an observation consistent with broader cultural analyses of gaming in the MENA region (Clément, 2019).

Each Saudi region represents a distinct social fabric marked by differences in tradition, urbanization, linguistic dialects, and access to digital infrastructure. These contextual features likely contribute to the emergence of regionally distinctive player profiles, extending prior findings that Saudi gamers engage with games in ways rooted in local identity and cultural archetypes (AlKhamees, 2023). In some areas, motivations were more aligned with competition, challenge, and progression, while others were more oriented toward narrative immersion, social connectedness, or creative expression. Such patterns suggest that gaming serves not only as a leisure activity but as a cultural practice through which regional values and lifestyles are negotiated, echoing earlier accounts of how gaming in Saudi Arabia functions as a site of identity expression (Balahmar, 2024).

Moreover, the relationship between regional identity and gaming behavior points to a more layered understanding of digital play, one that extends beyond global player typologies and acknowledges the ways local environments shape psychological engagement. Where players live may influence the types of games available to them, the communities they play with, and the expectations placed on their gaming behavior. This contextual shaping is likely reinforced by broader socio-cultural narratives, including family attitudes toward gaming (Almuhtadi & Alageel, 2025) and the availability of public gaming spaces (Arab News, 2018).

Importantly, this intra-national variation invites a rethinking of how cultural identity operates in digital contexts. Rather than viewing Saudi gamers as a homogenous group, the data support a more pluralistic model that recognizes regional diversity as a defining characteristic of gaming experience. This perspective not only enriches academic discussions of game cultures in the Middle East but also has practical implications for developers, marketers, and community managers seeking to engage players in a culturally resonant manner, complementing calls for culturally grounded localization practices (Al-Batineh & Alawneh, 2022; Jarrah et al., 2023). In this sense, gaming becomes a lens through which regional identity is expressed and reimagined, a site where local customs, aspirations, and social dynamics meet the global affordances of interactive media. Attending to these subtleties enables a more accurate and culturally grounded understanding of gaming as both a personal and place-based practice.

### 5.4. Practical and Theoretical Implications

The patterns revealed in this study suggest that gaming in Saudi Arabia functions as a space where gendered identities are continuously negotiated rather than merely expressed. Motivations such as immersion, sociality, or action do not simply map onto “men” or “women” preferences, but emerge from the cultural scripts players inhabit and the expectations they navigate while gaming. The stronger immersion and social orientations among Saudi women, for example, resonate with prior observation that gaming motivations are often shaped by gendered expectations of emotional expression and relationality (Lopez-Fernandez et al., 2019). Yet the Saudi context adds complexity, women reported high engagement, regular play, and interest in challenge-based genres, even as they strategically positioned themselves as “casual” gamers to avoid scrutiny. This suggests a form of identity management similar to what prior work by Lange et al. (2021) describe, where gendered labels are adopted or resisted depending on the social costs of occupying certain gameplay identities.

The regional interaction effects observed in Achievement, Mastery, and Immersion deepen this picture. Rather than a single national gaming culture, the findings point to regionally inflected subcultures in which gender norms are performed differently. The strong regional influence on mastery-driven motivations echoes earlier work showing that cultural context shapes the meaning and value of gameplay practices (AlKhamees, 2023; Balahmar, 2024). Gender and region appear to co-construct motivational landscapes. A woman from the Shamālī region and a woman from the Hijāzī region may both identify as casual gamers, but the cultural boundaries and opportunities surrounding their participation diverge. This layered configuration of identity, geography, and motivation complicates the universalist assumptions embedded in global models of gamer typologies such as Yee’s and emphasizes the need for context-aware interpretations of motivational data.

The experiences of harassment, linguistic stereotyping, and self-censoring behaviors give further weight to viewing gaming spaces as arenas where gender is actively negotiated. The reluctance to use microphones, the preference for anonymity, and the experience of gendered slurs show that players are not simply following their “preferences”, they are responding to a social environment in which visibility can carry risk. This mirrors findings by Ratan et al. and Wohn et al. that gendered digital spaces often push women to adopt protective or adaptive practices that shape their play styles and motivations (Ratan et al., 2021; Wohn et al., 2020). In the Saudi context, these adaptive practices are intensified through local cultural norms, family expectations, and region-specific dynamics of visibility. Thus, the motivations measured in this study can not be interpreted apart from the cultural labor required to inhabit gaming spaces safely.

The broader implication is that motivations are not only psychological dispositions but also socially produced responses to opportunities, constraints, and identity performance. Designing for Saudi gamers, therefore, requires attention to the cultural pressures players navigate: the need for safe communication environments, mechanisms that protect anonymity, and game features that affirm the legitimacy of diverse player identities. The preference for narrative depth and expressive play among women, for instance, may reflect a desire for spaces where identity can be explored with fewer social repercussions, echoing AlKhamees (2023) arguments that Saudi gamers use games as arenas for self-making and cultural negotiation. Similarly, the regional differences in achievement-oriented play suggest that localized gaming ecosystems, such as esports venues, public gaming cafés, and friend groups, shape how competitiveness is expressed.

Ultimately, the findings challenge the notion of a singular “Saudi gamer profile” and instead highlight a constellation of identities shaped by gender, identity, regional cultures, and social norms of play. These insights invite more research and design approaches that are attentive to the social fabric that shapes motivation, to the regional norms of play, and to the interactions and negotiations players engage in to inhabit digital worlds that are not always built with them in mind.

### 5.5. Implications Beyond the Saudi Context

Beyond the Saudi context, this research contributes to broader game user research by illustrating how intra-national cultural diversity can meaningfully shape player motivations. These findings suggest that future cross-cultural research would benefit from moving beyond national-level comparisons to consider subcultural and regional variation as a key analytical dimension. Doing so allows global motivation models to be tested more rigorously and interpreted more responsibly across diverse gaming populations.

When compared with studies from other cultural contexts, the findings of this study both align with and complicate existing cross-cultural patterns in gaming research. For instance, Khoshsaligheh and Ameri (2020) survey of Iranian gamers characterizes gaming as a predominantly male-oriented activity concentrated among younger players, with strong preferences shaped by national language and localization concerns. While our results similarly confirm the importance of cultural context in shaping gaming practices, the Saudi case diverges in notable ways. Unlike the Iranian sample, where women appear largely absent from gaming participation, Saudi women constitute a substantial and active segment of the gaming population, exhibiting distinct motivational profiles centered on social engagement and immersion rather than marginal or passive involvement. This contrast suggests that gendered access to gaming can not be uniformly explained by regional or religious proximity, but must be understood in relation to locally specific social transformations, digital access, and cultural negotiations.

Several of the motivational patterns identified in this study align with findings reported in research conducted in Europe, North America, and East Asia (AlKhamees, 2023; Balahmar, 2024; Jarrah et al., 2023; Khoshsaligheh & Ameri, 2020). Gender differences related to action-oriented play, mastery, and immersion mirror trends observed across multiple cultural contexts, suggesting that some gaming motivations reflect widely shared psychological tendencies or common forms of gendered socialization. These consistencies support the continued relevance of established motivation frameworks, such as Yee’s model, as useful tools for understanding player behavior across settings.

At the same time, the results demonstrate that the expression of these motivations is not uniform. The observed interaction effects between gender and region show that similar motivational tendencies can take different forms depending on local social norms, access to gaming spaces, and expectations surrounding participation. This finding complicates interpretations that rely solely on biological or universal explanations for gaming behavior and highlights the role of culture in shaping how motivations are experienced and enacted.

By examining regional variation within a single national context, this study offers a complementary approach to traditional cross-national research. Rather than treating countries as culturally homogeneous units, it shows how meaningful variation can exist within national borders. This perspective strengthens comparative game studies by encouraging more careful interpretation of cultural effects and by reducing the risk of overgeneralization when applying global models to local player communities.

Taken together, these implications suggest that gaming motivations should be understood as the result of an interaction between shared psychological drivers and culturally specific conditions. The Saudi case demonstrates how global patterns can coexist with local variation, reinforcing the value of context-aware approaches in games user research and cross-cultural studies.

## 6. Conclusions

This study provides a nuanced, data-driven portrait of gaming motivations among Saudi gamers, emphasizing the intersecting roles of gender, genre preferences, and geography. The findings reveal that gamer motivations in Saudi Arabia are neither homogenous nor solely driven by gender or geography alone, but rather shaped by their interplay. A key contribution of this work is its systematic examination of motivational dimensions across five major Saudi regions, something rarely attempted in prior literature. The study not only confirms well-established gender differences in gaming motivations, such as stronger action orientation in male gamers and higher social and immersion scores among women, but also uncovers regionally mediated differences in Achievement, Mastery, and Immersion motivations. Importantly, the findings challenge simplistic narratives about the “Saudi gamer” by illustrating that regional culture and social norms actively shape how and why individuals engage with games. These insights hold value not only for researchers but also for game developers, educators, and government entities interested in culturally responsive game design and development strategies in Saudi Arabia and beyond.

However, the study also presents several limitations. Although the overall sample size was robust and appropriate for the analyses conducted, the distribution of participants across certain demographic subgroups was uneven. This uneven subgroup representation may limit the generalisability of findings at the subgroup level and reduce statistical precision for those specific comparisons. Second, the study relied entirely on self-reported measures, which may be subject to recall bias or social desirability effects. Third, while motivation dimensions were measured quantitatively and rigorously, the research did not incorporate qualitative methods that might further contextualize how motivations manifest in everyday gaming practices. Future work could build on this study in several directions. First, follow-up studies should consider longitudinal approaches to explore how motivations evolve over time. Second, incorporating qualitative interviews, focus groups, or digital ethnographies could deepen the understanding of how regional and gendered identities are performed and negotiated through games. Third, expanding the scope beyond motivations to include gaming behaviors, social impacts, and psychological outcomes could provide a more holistic view of the Saudi gaming ecosystem. Finally, comparative studies across other Arab Gulf countries would offer valuable insight into whether the trends observed in this study are unique to Saudi Arabia or part of a broader regional phenomenon.

## Figures and Tables

**Table 1:** Demographic breakdown of participants by gender, age group, education level, self- reported gamer type, weekly play frequency, and region.

Variable	Category	Frequency	Percentage
Gender	Women	247	62.22
	Men	150	37.78
Age	18–24	170	42.82
	35–44	102	25.69
	25–34	62	15.62
	45–54	58	14.61
	55–64	6	1.26
Education	Bachelor’s	199	50.13
	High School	132	33.25
	Diploma	42	10.58
	Master’s	15	3.78
	PhD	9	2.27
Gamer Type	Hobbyist	102	25.69
	Casual	212	53.4
	Serious	83	20.91
Play per Week	0–1 Days	154	38.79
	2–3 Days	101	25.44
	4–5 Days	52	13.1
	6–7 Days	90	22.67
Regions	Western	139	35.01
	Central	89	22.42
	Eastern	72	18.14
	Southern	51	12.85
	Northern	46	11.59

## Data Availability

The original contributions presented in this study are included in the article. Further inquiries can be directed to the author.

## Supplementary Materials

The following supporting information can be downloaded at: https://www.mdpi.com/article/10.3390/bs16020202/s1.
