# Supplementary material for: What Drives Saudi Gamers? A Study of Gender, Genre, and Geography
Source: Behav Sci (Basel). 2026 Jan 30;16(2):202. doi: 10.3390/bs16020202 (PMC12938362; doi:10.3390/bs16020202)
Supplement: Supplementary file 1 [file behavsci-16-00202-s001.zip › behavsci-4017211-supplementary.pdf]

Understanding Gamers in Saudi Arabia

Nick Yee Gamer Motivation Profile Survey Instrument

What is your age?

- Under 18
- 18–24
- 25–34
- 35–44
- 45–54
- 55–64
- 65 and above

What is your gender?

- Man
- Woman
- Prefer not to disclose

Are you a Saudi citizen?

- Yes
- No

Which region in Saudi Arabia do you live in?

- Riyadh Region
- Makkah Region
- Madinah Region
- Qassim Region
- Eastern Province
- Asir Region
- Tabuk Region
- Hail Region
- Northern Borders
- Jazan Region
- Najran Region
- Al Baha Region
- Al Jouf Region

Educational level

- Primary
- Intermediate
- Secondary
- Diploma
- Bachelor’s degree
- Higher diploma
- Master’s degree
- Doctorate

How many days per week do you spend playing video games?

- 0–1 day
- 2–3 days
- 4–5 days
- 6–7 days

How would you describe yourself?

- Casual gamer (you play games but in short or infrequent sessions)
- Hobbyist gamer (you play regularly but without seriousness or competitiveness)
- Serious gamer (you are dedicated and play seriously or competitively)

Which platforms do you usually play on? (Select all that apply)

- Console (Xbox, PlayStation, Nintendo)
- PC/Mac (desktop or laptop)
- Smartphone/tablet (iOS, Android)
- Handheld consoles (Nintendo Switch, PS Vita)
- Virtual Reality (Oculus Quest/Rift, PlayStation VR)

Are you currently playing any video games?

- Yes
- No

What types of video games do you enjoy playing? (Select all that apply)

- Action
- Adventure
- Role-Playing Games (RPG)
- Sports
- Puzzle
- Simulation
- Strategy
- First-Person Shooter
- Multiplayer
- Open World
- Other (please specify)

Provide examples of video games you enjoy

(Free text)

Do you play tabletop or card games such as Baloot or Basra?

- Yes
- No

Provide examples of tabletop/card games you enjoy

(Free text)

How important are the following elements of a video game to you?

Rated on a scale from “Not important” to “Very important”:

- A well-crafted story
- Collecting large amounts of in-game resources or currency
- Taking time to master the game
- Getting strong and unique weapons
- Making an effort to acquire all collectible items
- Role-playing or pretending to be another character
- Having characters with interesting backstories
- Becoming as powerful as possible in the game
- Getting to know all the main characters and their backgrounds
- Completing all missions and achievements
- Dominating other players
- Earning every star/trophy/achievement
- Playing at the highest difficulty
- Discovering new or unconventional ways to play
- Having many customization colors, skins, and styles

How much do you enjoy the following?

Rated from “Not enjoyable at all” to “Very enjoyable”:

- Facing difficult challenges requiring many attempts
- Causing chaos or destruction in the game
- Helping other players
- Facing other players in matches or duels
- Long-term strategic planning
- Fast-paced gameplay requiring intense planning
- Blowing things up
- Taking on the role of a different character
- Immersing yourself in another world or setting
- Using weapons and explosives
- Working with other players toward a shared goal
- Gameplay requiring quick reactions
- Gameplay requiring a lot of thinking and planning
- Games with a lot of blood
- Gathering with other players in-game
- Competing with other players

How often do you do the following while gaming?

Rated from “Never” to “Always”:

- Explore the game world just to explore
- Focus mainly on increasing your stats/level
- Spend a lot of time customizing characters/cities/environments
- Study other players to improve your strategy
- Experiment with different things to see what the game world allows
- Communicate with others

Answer these open-ended questions:

- What motivates you to play video games?
- Do you prefer playing alone, with friends, or with strangers online? And why?
- Do you feel comfortable using a microphone or revealing your identity in video games? Why or why not?
- Do you feel safe when playing online video games with strangers? And why?
- Have you experienced any criticism, bullying, or uncomfortable situations because you are a gamer? Can you share a specific incident?
- Does your family support your gaming habits, and what is their view of video games in your opinion?
